# Simvastatin reduces high uric acid-induced oxidative stress and inflammatory response in vascular endothelial cells via nuclear factor E2-related factor 2 (Nrf2) signaling

**DOI:** 10.22038/IJBMS.2023.69187.15074

**Published:** 2023

**Authors:** Xuemeng Chen, Li Xie, Wei Wu

**Affiliations:** 1 Department of Traditional Chinese Medicine and Rheumatism Immunology, The First Affiliated Hospital of Army Medical University, Chongqing 400038, P.R. China; 2 Traditional Chinese Medicine Department, The People’s Hospital of Dadukou District Chongqing, Chongqing, 400084, P.R. China

**Keywords:** Inflammation, Nrf2 signaling, Oxidative stress, Simvastatin, Uric acid, Vascular endothelial cells

## Abstract

**Objective(s):**

Increased oxidative stress and inflammatory response are risk factors for kidney and cardiovascular diseases in patients with hyperuricemia. Uric acid (UA) has been reported to cause inflammation and oxidative damage in cells by inhibiting the nuclear factor E2-related factor 2 (Nrf2) pathway. Notably, Simvastatin (SIM) can regulate the Nrf2 pathway, but whether SIM can regulate inflammatory response and oxidative stress in vascular endothelial cells induced by high UA via this pathway has not been clarified.

**Materials and Methods:**

To demonstrate this speculation, cell activity, as well as apoptosis, was estimated employing CCK-8 and TUNEL, respectively. Indicators of oxidative stress and inflammation were assessed by related kits and western blotting. Subsequently, the effects of SIM on signaling pathways were examined using western blotting.

**Results:**

The result showed that after UA exposure, oxidative stress was activated and inflammation was increased, and SIM could reverse this trend. Meanwhile, SIM could inhibit high UA-induced apoptosis. In addition, western blotting results showed that SIM reversed the down-regulation of the expression of Nrf2 pathway-related proteins caused by high UA.

**Conclusion:**

SIM alleviated the inflammatory response as well as inhibiting oxidative stress through the Nrf2 pathway, thereby attenuating high UA-induced vascular endothelial cell injury.

## Introduction

Hyperuricemia is caused by decreasing or excessive excretion of uric acid (UA) (1, 2). Previous studies have found that hyperuricemia is associated with increased risk of gout, kidney, and cardiovascular diseases (2-4). Notably, UA is considered a risk signal within cells and the end product of purine metabolism (5). Persistent high expression levels of UA can lead to production of urate crystals in the articular cavity. Meanwhile, soluble urates or urate crystals can induce inflammatory response as well as oxidative stress, which are responsible for disease advancement (4-7). Thence, research on the mitigation of oxidative stress and inflammation following high UA (HUA) stimulation is of critical importance. 

Statins have been extensively applied in clinical trials due to their numerous effects in addition to lipid-lowering effects (8). They provide direct anti-oxidant effects by clearing free radicals and activating anti-oxidant enzymes (9). At the same time, a rising number of studies have shown that statins, which serve as modulators of the expression of cytokines and proteins, have anti-oxidant and anti-inflammatory effects. For example, Simvastatin (SIM) is a representative drug among statins. According to the literature, SIM could attenuate diabetic cardiomyopathy by reducing hyperglycemia/hyperlipidemia-induced oxidative stress and inflammation (10); and SIM could alleviate lipopolysaccharide-stimulated inflammatory and oxidative responses in alveolar macrophages (11). Additionally, SIM could reduce inflammatory damage as well as oxidative stress via the Nrf2-ARE pathway and exert neuroprotective effects against traumatic brain injury (12). These studies demonstrated the role of SIM in preventing pathological inflammation and oxidative stress. Therefore, it is hypothesized that SIM may impact hyperuricemia-induced oxidative stress and inflammatory response and may be a potential drug for the treatment of hyperuricemia.

However, the protective effect of SIM on vascular endothelial cells has not yet been reported. Nuclear factor erythroid 2-related factor 2 (Nrf2), which is a redox-sensitive transcription factor, is the central control factor of NADH Dehydrogenase, Quinone 1 (NQO1) expression under normal homeostasis and oxidative stress (13, 14). Extracellular oxidant stress stimulates the phosphorylation and dissociation of Nrf2 from Keap1. Subsequently, the accumulation of Nrf2 in the nucleus results in nuclear gene translocation and high expression of its downstream anti-oxidant enzyme Heme oxygenase 1 (HO-1), thus playing anti-inflammatory and anti-oxidant roles (15). 

This study was designed to investigate the possible role of SIM through the Nrf2 signaling pathway in an HUA-induced vascular endothelial cell model.

## Materials and Methods

***Cell culture***

The human umbilical vein endothelial cells (HUVECs) provided by American Type Culture Collection were plated in endothelial cell medium which was supplemented with 5% FBS, 1% penicillin/streptomycin solution, as well as 1% endothelial cell growth addition (Solarbio Biotechnology Co., Ltd) and was placed at 37 °C with 5% CO_2_ (16). An *in vitro* model of hyperuricemia was established by stimulation with 100 μg/ml UA (HY-B2130, MedChemExpress), and subsequently, HUVECs were administrated with 0.2 μM SIM (HY-17502, MedChemExpress). ML385 (10 μm, HY-100523, MedChemExpress), an Nrf2 inhibitor, was used to pretreat the cells for one hour which were then cultured with UA and/or SIM for 24 hr.

***Cell vitality assay***

The inoculation of HUVECs in 96-well plates was implemented for cell cultivation with UA and/or SIM for 24 hr. Subsequently, 10 μl cell counting kit-8 (CCK-8) solution (Shanghai Fusheng Industrial Co, Ltd) was put into every well to further foster the cells. With the aid of an enzyme marker (Hangzhou Wanuo Biotechnology Co, Ltd.), the absorbance was tested on the premise of λ = 450 nm (17).

***Detection of inflammatory cytokines***

To determine the levels of inflammatory cytokines, ELISA was applied. The levels of interleukin-1β (IL-1β), as well as interleukin-18 (IL-18), were examined with an ELISA kit (Elabscience Biotechnology Co., Ltd) in light of standard protocol.

***Measurement of oxidative stress indicators***

The activity of oxidative stress indicators reactive oxygen species (ROS), superoxide dismutase (SOD), glutathione peroxidase (GSH-Px), catalase (CAT), as well as malondialdehyde (MDA) in HUVECs was tested employing related kits (Nanjing Jiancheng Bioengineering Institute Co, Ltd) in line with standard specifications.

***Western blotting assay***

The quantification of proteins that were isolated from indicated cells adopting RIPA buffer was operated using a BCA protein assay kit. After subjection to 10% SDS-PAGE, the transfer of proteins to PVDF membranes was carried out. The overnight exposure of membranes that were impeded by 5% skim milk to anti-Bcl-2 (ab32124; 1:1000; Abcam), anti-Bax (ab32503; 1:1000; Abcam), anti-Nrf2 (ab62352; 1:1000; Abcam), anti-NQO-1 (ab80588; 1:10000; Abcam), anti-HO-1 (ab52947; 2:1000; Abcam), anti- glutathione peroxidase 1 (GPX1, ab108427; 2:1000; Abcam), or anti-GAPDH (ab6759; 1: 5000; Abcam) was implemented at 4 °C, after which the probe with proper secondary antibody was used. The visualization of protein blots was tracked utilizing the ECL kit (Nanjing Novozymes Biotechnology Co, Ltd), and the analysis was conducted with the application of Image-Pro Plus version 6.0 software (Roper Technologies, Inc.) (18).

***TUNEL assay***

Terminal Deoxynucleotidyl Transferase mediated dUTP Nick-End Labeling (TUNEL) was adopted for the estimation of apoptotic cell death. Initially, cells were subjected to fixation with 3.7% paraformaldehyde as well as permeabilization with 0.1% Triton X-100. Subsequently, the cultivation of samples with TUNEL assay solution was implemented under light-proof conditions at 37 °C for one hour. Following nuclear staining with DAPI and addition of an anti-fading solution, all samples were photographed at random for observation adopting a fluorescence microscope (magnification, x100) (19).

***Reverse-transcriptase-polymerase chain reaction (RT-PCR)***

Total RNA was isolated from the cells with Trizol (Invitrogen USA). Revert Aid (TM) first strand cDNA synthesis kit (Ferments Life Science, Fort Collins, CO, USA) was used for RNA reverse transcription. The sequences were as follows: Tumor necrosis factor alpha (TNF-α) sense: 5′-CTGGGCAGGTCTACTTTGGG-3′, antisense: 5′- CTGGAGGCCCCAGTTTGAAT-3′; IL-18 sense: 5′- ATCGCTTCCTCTCGCAACAA-3′, antisense: 5′- GAGGCCGATTTCCTTGGTCA-3′; IL-1β sense: 5′- CCAAACCTCTTCGAGGCACA -3′, antisense: 5′- AGCCATCATTTCACTGGCGA -3′; IL-6 sense: 5′-GTCCAGTTGCCTTCTCCCTGG-3′, antisense: 5′- CCCATGCTACATTTGCCGAAG-3′; GAPDH sense: 5′- AATGGGCAGCCGTTAGGAAA-3′, antisense: 5′- GCGCCCAATACGACCAAATC-3′.

***Statistical analysis***

Data displayed in the form of mean ± SD was analyzed with GraphPad Prism 8.0 software (GraphPad Software, Inc.). Comparisons among different groups were demonstrated by one-way ANOVA as well as Tukey’s test. *P-*values*<0.05* meant statistical significance. All experiments were repeated more than 3 times.

## Results

***Determination of the application doses of UA and SIM***

**Figure 1:**
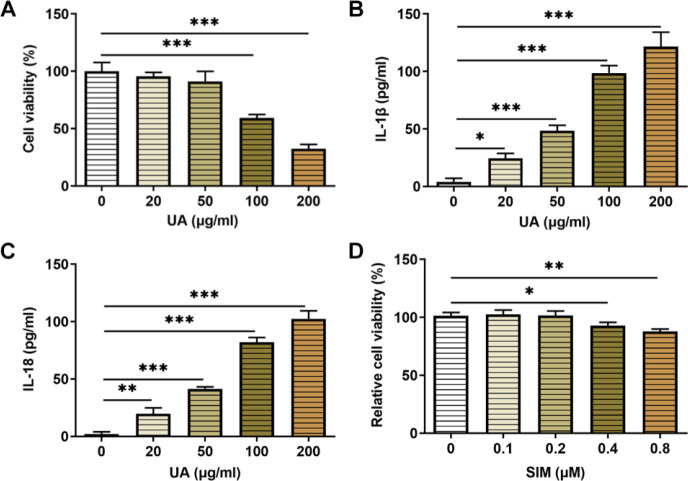
Establishment of a HUA-induced HUVECs model. (A) Effect of UA on the viability of HUVECs by CCK-8 assay. n=5. Expressions of (B) IL-1β and (C) IL-18 in HUVECs by ELISA assay. n=5. (D) Effect of SIM on the activity of HUVECs by CCK-8 assay. n=5. *P<0.05, **P<0.01, and ***P<0.001

HUVECs: Human umbilical vein endothelial cells; HUA: Inflammation following high UA; UA: Uric acid; CCK-8: Cell counting kit-8; IL: Interleukin

The application doses of UA and SIM were determined by examination of HUVECs activity. The CCK-8 results showed that the cell viability was 59% and 32% when treated by UA at concentrations of 100 μg/ml and 200 μg/ml, individually, both of which greatly differed from that in the control group (Figure 1A). Meanwhile, UA raised the levels of IL-1β as well as IL-18 in HUVECs in a dose-dependent way (Figure 1B-1C). Therefore, UA at a concentration of 100 μg/ml was chosen for follow-up experiments. It was found that no changes were noticed in cell activity upon exposure to 0.2 μM SIM, but significant damage was caused upon exposure to 0.4 μM when compared with the control group (Figure 1D). Therefore, SIM at a concentration of 0.2 μM was chosen for the ensuing experiments.

***SIM inhibits oxidative stress and inflammation in HUVECs induced by HUA***

**Figure 2:**
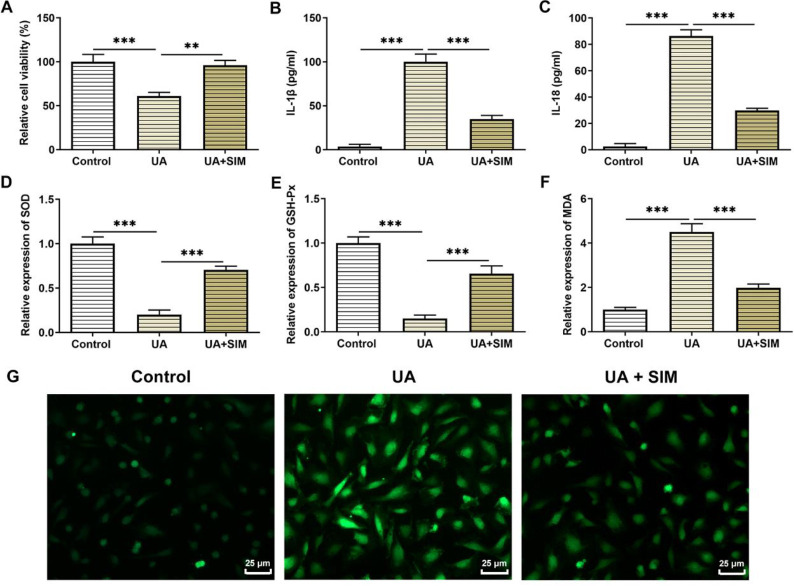
Effects of SIM on oxidative stress and inflammation in HUA-induced HUVECs. (A) Viability of HUVECs was detected by CCK-8 assay. n=5. Expressions of (B) IL-1β and (C) IL-18 in HUVECs by ELISA assay. n=5. Results of oxidative stress include the levels of (D) SOD, (E) GSH-Px, and (F) MDA. n=5. Content of ROS (G) in HUVECs by corresponding assay kit. n=3. Magnification×400. *P<0.05, **P<0.01, and ***P<0.001

SIM: Simvastatin; HUA: Inflammation following high UA; HUVECs: Human umbilical vein endothelial cells; CCK-8: Cell counting kit-8; IL-18: Interleukin-18; MDA: Malondialdehyde

An *in vitro* model of hyperuricemia was established by stimulation with 100 μg/ml UA, and subsequently, HUVECs were administrated with 0.2 μM SIM. The cell activity results showed that co-administration of UA and SIM significantly increased cell activity when compared with that in cells induced by HUA (UA group) (Figure 2A). At the same time, HUA exposure remarkably enhanced the levels of IL-1β and IL-18, however, in contrast, SIM inhibited the increase in the expressions of these inflammatory factors (Figure 2B-2C). The oxidative indicators (SOD, GSH-PX, and MDA) were evaluated. The results demonstrated that SIM could back-regulate the decreased SOD and GSH-Px expressions as well as increased MDA expression due to HUA exposure (Figure 2D–2F). As depicted in Figure 2G, UA greatly enhanced ROS content in contrast with that in the control group, which was then reduced by SIM administration.

***SIM inhibits apoptosis in HUVECs induced by HUA***

**Figure 3:**
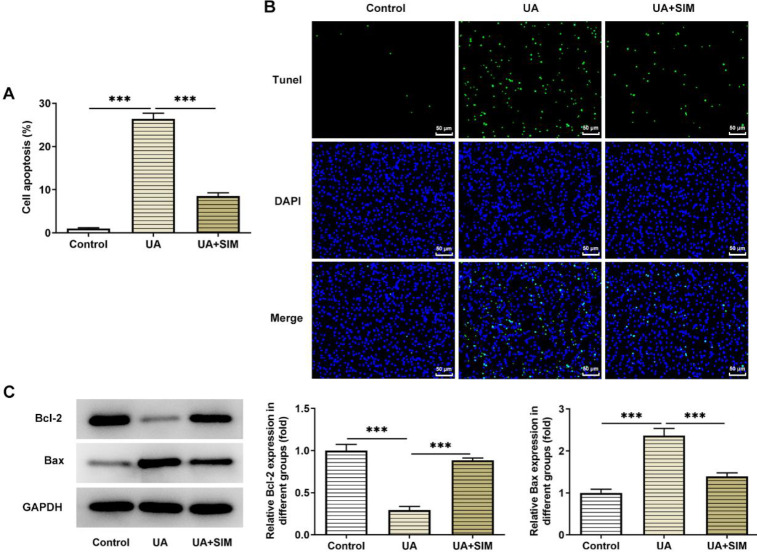
Effects of SIM on the apoptosis of HUA-induced HUVECs. (A and B) Representative apoptosis images by TUNEL staining. n=3 Magnification×200. (B) Expression of apoptosis-related proteins by western blotting. n=3. *P<0.05, **P<0.01, and ***P<0.001

SIM: Simvastatin; HUA: Inflammation following high UA; HUVECs: Human umbilical vein endothelial cells; TUNEL: Transferase mediated dUTP Nick-End Labeling

Results obtained from TUNEL suggested that in contrast with the control group, HUVECs apoptosis in the UA group was prominently exacerbated (Figures 3A and B). However, apoptosis was significantly reduced in HUVECs in the UA+SIM group compared with the UA group. The expression of apoptosis-related proteins was assessed employing western blotting (Figure 3C). In the UA group, Bcl-2 level declined, while Bax, cleaved-caspase3, and cleaved-caspase9 levels were elevated in contrast with that in the control group. However, Bcl-2 expression was increased while Bax, cleaved-caspase3, and cleaved-caspase9 expression were decreased in the treatment group (UA+SIM group) compared with the UA group.

***SIM activates the Nrf2 pathway in HUVECs induced by HUA***

**Figure 4:**
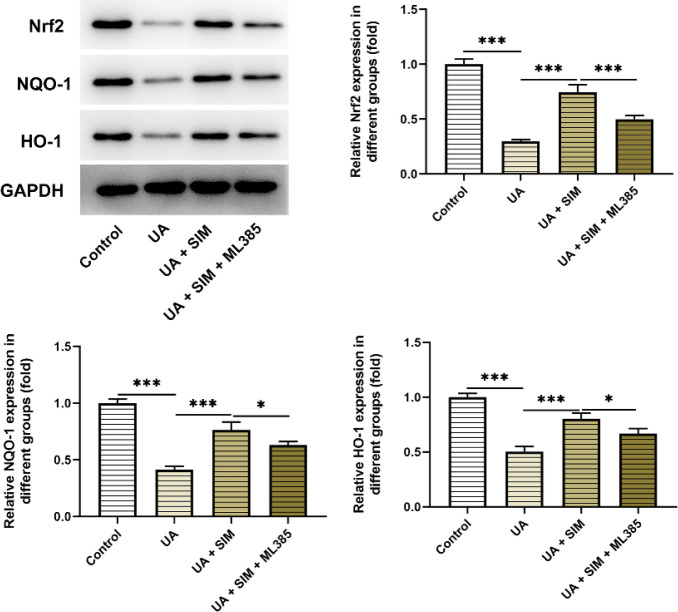
Western blotting was used to detect the expression of Nrf2 signaling pathway-related proteins Nrf2, NQO-1, and HO-1 affected by SIM. n=3. *P<0.05, **P<0.01, and ***P<0.001

Nrf2: Nuclear factor E2-related factor 2; NQO-1: NADH Dehydrogenase, Quinone 1; HO-1: Heme oxygenase 1; SIM: Simvastatin

The effects of UA and SIM on the Nrf2 signaling pathway were then investigated (Figure 4). In contrast with the Control group, UA conspicuously declined the contents of Nrf2, NQO-1, and HO-1, while SIM administration imparted opposite impacts on these proteins, evidenced by the elevated contents of Nrf2, NQO-1, and HO-1 in UA +SIM group, implying that SIM partially abolished the suppressive effects of UA on the Nrf2 signaling pathway. Additionally, the stimulatory role of SIM was counteracted by ML385, testified by diminished contents of Nrf2, NQO-1, and HO-1 in UA+ SIM + ML385 group by contrast with the UA +SIM group. Thus, SIM might reduce HUA induced HUVECs injury by regulating the Nrf2 signaling pathway.

***SIM inhibits oxidative stress, inflammation, and apoptosis by regulating the Nrf2 pathway***

**Figure 5:**
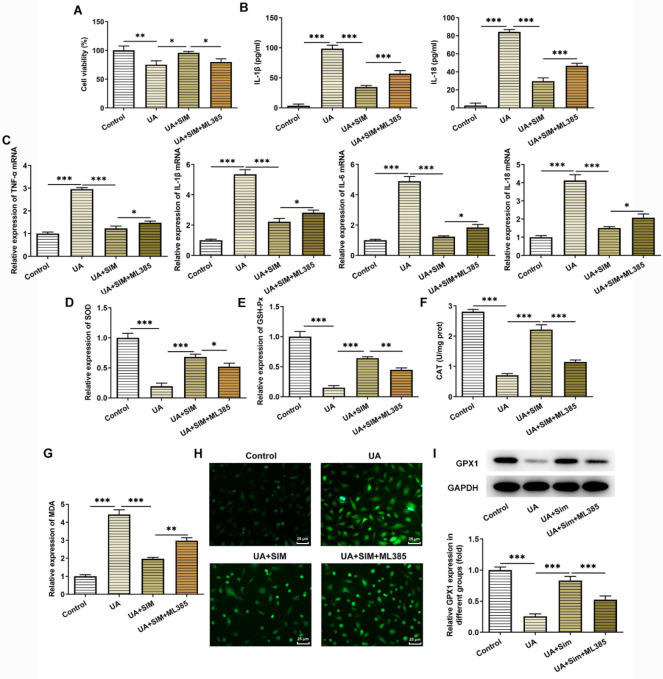
SIM inhibits oxidative stress and inflammation by regulating the Nrf2 pathway. (A) Viability of HUVECs was detected by CCK-8 assay. n=5. (A) ELISA was used to detect the expression of IL-1β and IL-18 in HUVECs induced by high UA. (B) RT-qPCR detected the expression of TNF-α, IL-1β, IL-6, and IL-18. n=5. Detection of the levels of oxidative stress indicators including (C) SOD, (D) GSH-Px, (E) CAT, and (F) MDA. n=5. (G) Detection of ROS level. n=3. Magnification×400. (H) Western blotting analysis of GPX1 protein. n=3. *P<0.05, **P<0.01, and ***P<0.001

SIM: Simvastatin; Nrf2: Nuclear factor E2-related factor 2; CCK-8: Cell counting kit-8; IL: Interleukin; SOD: Superoxide dismutase; TNF-α: Tumor necrosis factor alpha; GSH-Px: Glutathione peroxidase; MDA: Malondialdehyde; ROS: reactive oxygen species

CCK8 results showed that compared with the UA+SIM group, cell viability was decreased (Figure 5A). The results of ELISA showed that ML385 administration significantly increased the expression levels of TNF-α, IL-1β, IL-6, and IL-18 (Figures 5B and C), indicating that SIM could inhibit inflammatory response by activating the Nrf2 pathway. Compared with the UA+SIM group, ML385 administration enhanced the MDA level but declined the levels of SOD, CAT, and GSH-Px (Figure 5D–G). As Figure 5H demonstrated, the enhanced ROS content in HUA-induced HUVECs was reduced by SIM in comparison with that in the UA group, which was then partially elevated after ML385 administration. Subsequently, the expression of oxidative stress-related selenoprotein GPX1 was detected, and its trend was consistent with that of SOD (Figure 5I). 

**Figure 6:**
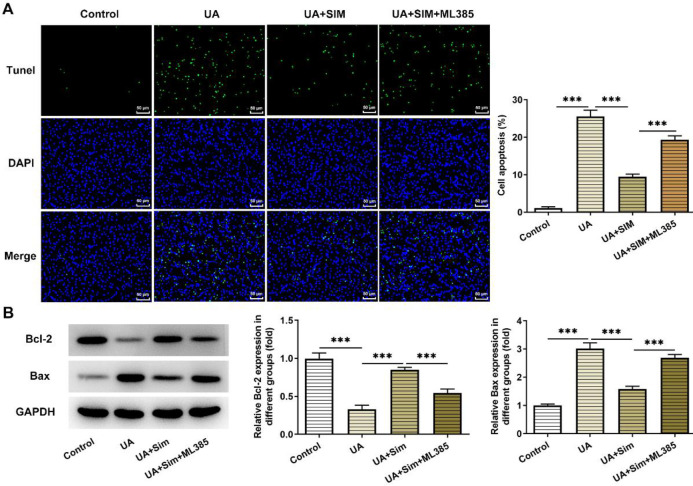
(A) Representative apoptosis images by TUNEL staining and the results of HUVECs apoptosis rate. n=3. Magnification×200. (B) Expression of apoptosis-related proteins by western blotting. n=3. ***P<0.001

TUNEL: Transferase mediated dUTP Nick-End Labeling; HUVECs: Human umbilical vein endothelial cells

Additionally, results obtained from TUNEL demonstrated that in contrast with the UA+SIM group, the inhibitory effects of SIM on apoptosis were greatly diminished by inhibition of the Nrf2 pathway by ML385 (Figure 6A). And results in Figure 6B showed that the content of Bax was hugely increased due to ML385, while Bcl-2 was remarkably reduced in comparison with UA+SIM group.

## Discussion

Hyperuricemia is produced by high levels of UA *in vivo* (2). HUA has been reported to elicit detrimental activities by promoting oxidative stress caused by reactive oxygen species (ROS) components (20). Notably, oxidative stress where there is an imbalance between oxidants (or ROS) and anti-oxidative systems *in vivo* (21) has been considered to be an important cause of diseases and cancers, such as cardiovascular diseases (22), diabetes (23), breast cancer, lung cancer (24-26), as well as inflammation (27). More importantly, the concurrent emergence of inflammation and oxidative stress greatly increases the risk of diseases (28). Thus, detection of oxidative stress and inflammatory response is essential in the therapy for hyperuricemia.

In the present study, the HUA-induced hyperuricemia model was successfully constructed in HUVECs. Subsequently, the effect of SIM on HUA-induced HUVECs was evaluated by the changes in oxidative stress levels and the release of inflammatory cytokines. The results showed that SIM conspicuously repressed apoptosis as well as the levels of IL-1β and IL-18 in HUVECs. Besides, SIM also protected against HUA-elicited oxidative stress, thus mitigating HUA-induced injury in HUVECs.

Subsequently, the downstream mechanism of SIM was investigated. Nrf2 acts as a key transcription factor in regulating cellular responses to oxidative stress and inflammation (29). In another way, a summary review revealed that Nrf2 was a pro-survival factor and a main regulator of cytoprotective mechanisms, while Nrf2-ARE signaling could modulate cellular response under oxidative stress (30). Meanwhile, further studies proved that drugs might protect against oxidative stress and mitochondrial abnormalities due to hyperuricemia via the Nrf2 signaling pathway (31). To substantiate the effect of SIM on oxidative stress and inflammatory factors through the Nrf2 signaling pathway, further investigations were conducted in this study.

Notably, SIM, which is a typical statin with good tolerability, has been increasingly reported (8). It was shown that SIM reduced hyperglycemia and hyperlipidemia while reducing oxidative stress and enhancing anti-oxidant defense in a rat model of diabetes (10). This report also demonstrated that SIM had a strong regulatory effect on inflammation and apoptosis. Additionally, Zhang *et al*. found that SIM could attenuate TLR4-mediated inflammatory damage and defend against oxidative stress through the Nrf2 signaling pathway in a rat traumatic brain injury model, thus playing a neuroprotective role against traumatic brain injury (12). The previous study showed that SIM protected human melanocytes from H2O2-induced oxidative stress by activating Nrf2 (32). These studies indicated that SIM could directly act on the Nrf2 signaling pathway and demonstrated the value of SIM in alleviating pathological inflammation and oxidative stress. Therefore, we hypothesized that SIM played a regulatory role in oxidative stress and inflammatory response induced by hyperuricemia, and could serve as a potential drug for the improvement of hyperuricemia. 

Subsequently, the proteins related to Nrf2 signaling pathway were detected. And the results demonstrated that SIM could enhance the contents of Nrf2, NQO-1, as well as HO-1 in HUVECs induced by HUA, which was in line with the previous finding that SIM alleviated oxidative stress-induced renal ischemia/reperfusion injury by targeting the Nrf2/HO-1 pathway (33). Meanwhile, it was also discovered that the Nrf2 level was repressed, which further confirmed that SIM might regulate biological processes through Nrf2 pathways. The findings implied that SIM imparted suppressive impacts on oxidative stress, inflammatory response, and apoptosis in HUA-induced HUVECs by activating the Nrf2 pathway. 

Nevertheless, there are also some limitations in this work. Other possible signaling pathways that may be regulated by SIM will be further discussed in future experiments. In addition, other cell lines are needed to further verify our experimental results in addition to HUVECs. 

## Conclusion

In short, the present study further supported the potential value of SIM for the treatment of hyperuricemia.

## Authors’ Contributions

W W and X C conceived and designed the study. X C and L X performed experiments. W W wrote the paper. W W, X C, and L X reviewed and edited the manuscript. W W, X C, and L X confirm the authenticity of all the raw data. All authors have read and approved the final manuscript.

## Highlights

SIM reduces high uric acid-induced oxidative stress in vascular endothelial cells. SIM reduces the high uric acid-induced inflammatory response in vascular endothelial cells. SIM activates the Nrf2 signaling pathway.

## Ethics Approval and Consent to Participate

This article does not cover animal experiments and human experiments, thus ethical approval is not required.

## Funding

Personal funds from authors.

## Conflicts of Interest

The authors declare that they have no competing interests.
